# Seeking Sense in the Hox Gene Cluster

**DOI:** 10.3390/jdb10040048

**Published:** 2022-11-15

**Authors:** Stephen J. Gaunt

**Affiliations:** Department of Zoology, University of Cambridge, Downing Street, Cambridge CB2 3EJ, UK; sg397@cam.ac.uk

**Keywords:** Hox cluster, collinearity, evolution, axial morphology, gene knockout, Bilateria, Cnidaria

## Abstract

The Hox gene cluster, responsible for patterning of the head–tail axis, is an ancestral feature of all bilaterally symmetrical animals (the Bilateria) that remains intact in a wide range of species. We can say that the Hox cluster evolved successfully only once since it is commonly the same in all groups, with *labial*-like genes at one end of the cluster expressed in the anterior embryo, and *Abd-B*-like genes at the other end of the cluster expressed posteriorly. This review attempts to make sense of the Hox gene cluster and to address the following questions. How did the Hox cluster form in the protostome-deuterostome last common ancestor, and why was this with a particular head–tail polarity? Why is gene clustering usually maintained? Why is there collinearity between the order of genes along the cluster and the positions of their expressions along the embryo? Why do the Hox gene expression domains overlap along the embryo? Why have vertebrates duplicated the Hox cluster? Why do Hox gene knockouts typically result in anterior homeotic transformations? How do animals adapt their Hox clusters to evolve new structural patterns along the head–tail axis?

## 1. Introduction

**Figure 1:**
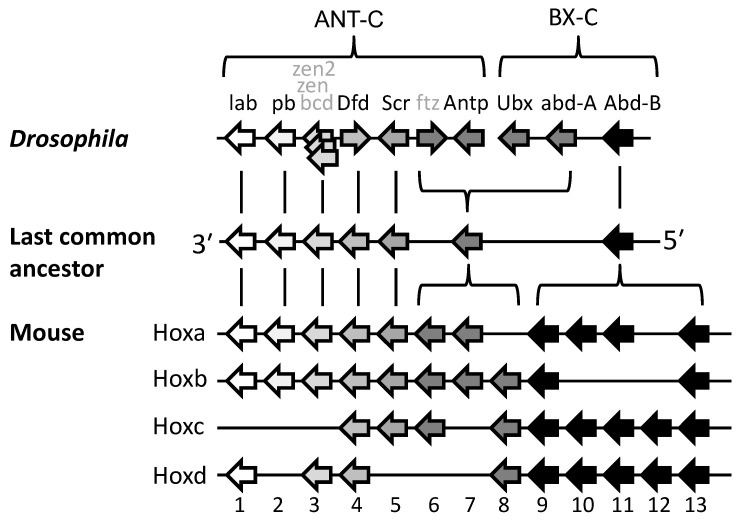
Homologous Hox gene clusters in *Drosophila*, mouse/human and, by inference, their common ancestor. The ancestor (the protostome–deuterostome last common ancestor, P-DLCA) may have had more than the seven genes shown here [4]. Genes that share the same numbers and shading intensities are most recently related by descent. Hox-derived genes in *Drosophila* that no longer function as true Hox genes are named in grey text. Arrows indicate directions of transcription (presumed for ancestor). ANT-C, Antennapedia complex; BX-C, Bithorax complex.

An unexpected discovery in the 1980s was that both arthropods (*Drosophila*) and vertebrates (mice) utilise conserved clusters of Hox genes in order to specify pattern formation along their head–tail axes. The widely held conclusion is that the common ancestor to these two groups (the protostome–deuterostome last common ancestor, or P-DLCA) already possessed a cluster of seven, or more, Hox genes which it used, most likely as in its descendants today, for specification of distinct body parts along the anterior-to-posterior (A-P) axis (Figure 1) [1,2,3].

The Hox cluster is an ancestral feature of all bilaterally symmetrical animals (Bilateria) retained in many, though not all, species (Section 2.2). We can say that it evolved successfully only once since the cluster is the same in all groups, with *labial*-like genes at one end of the cluster expressed in the anterior embryo, and *Abd-B*-like genes at the other end of the cluster expressed posteriorly [4,5,6].

Although now disrupted in some descendants, the Hox gene cluster in many other descendants, both protostome and deuterostome, is still conserved to a large extent in its inferred ancestral form [4,5,6]. The entire cluster has undergone duplications in vertebrates (Figure 1, Section 2.6) though it remains unduplicated in invertebrate deuterostomes [4,5,6].

**Figure 2:**
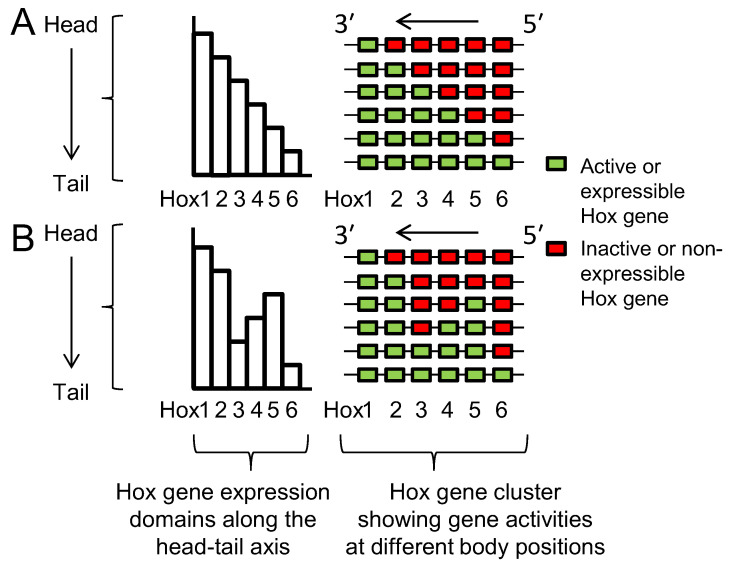
Hox expression patterns which do, or do not, conform to Lewis’s collinearity model. (**A**) Conforming to Lewis’s model for *Drosophila* [7], and as now known to apply in many different animals [5,6], Hox genes are expressed in a series of partially overlapping domains along the body with the order of genes along the cluster being collinear with the order of their expression domains along the head–tail axis. This is shown in Figure 2A-left, and the genes active at different positions along the body are shown in Figure 2A-right. (**B**) Scenario where *Hox3* and *Hox5* gene expressions do not conform to collinearity. Arrows show directions of transcription. Figure from Gaunt 2015 [8].

From his pioneering analyses of *Drosophila* developmental mutants, Ed Lewis [7] was the first to propose the following (Figure 2A): that the clustered set of genes now known as Hox genes are expressed in a series of partially overlapping domains along the length of the embryo; that the order of the genes along the chromosome corresponds with the order of their expression domains along the head–tail axis (the spatial collinearity rule); and that the blend of Hox genes active in each segment or A-P domain of the body is responsible for the specification of its structure. Subsequent work showed that these predictions hold true for many or most bilaterally symmetrical animals (Section 2.4, Section 2.7 and Section 2.8).

**Figure 3:**
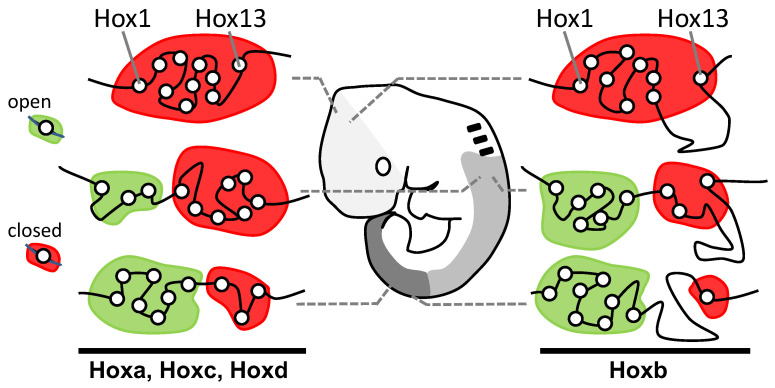
Discreet domains of open and closed chromatin generally support Lewis’s model. Antibody studies show correspondence between the position of cells along the head–tail axis and the distributions of Hox genes between open (Hox expressible, green domain; Trx-rich) and closed (Hox non-expressible, red domain; Pc-rich) chromatin states. At each level along the body, there is only a single boundary between these states, supporting Lewis’s model (Figure 2A-right). Re-drawn/modified from Noordermeer et al. [10].

Lewis’s model describes Hox genes as expressing or non-expressing, but we now understand that these states are more accurately described as, respectively, expressible or non-expressible. Recent studies in both mice [9,10] and *Drosophila* [11] show that the expressible Hox genes in a given cell (Figure 2A-right) are in an open chromatin state, characterized by Trithorax (Trx) protein binding, while the non-expressible genes are in a closed chromatin state, characterized by Polycomb (Pc) protein binding (Figure 3). These chromatin states are usually heritable from one cell generation to the next, thereby ensuring that Hox expressibility patterns acquired in the early embryo are faithfully remembered at all later stages, enabling guidance throughout the course of development. At any region along the body, there is typically only one boundary between expressible and non-expressible genes in both mice [9,10] (Figure 2A-right and Figure 3) and *Drosophila* [11]. In the subtle difference from Lewis’s original proposal, expressible genes need not always be expressed, as illustrated in Section 2.4 and Section 2.8. Expression depends upon the availability of activating or repressive transcription factors and may change in a tissue over developmental time. Expressible Hox genes have been described as “open for business” [12,13]. Regions of the body where Hox genes are non-expressible (closed for business; red zones in Figure 3) typically cannot express these genes at any time.

In addition to spatial collinearity in Hox expression, many species including vertebrates [14], cephalochordates [15], some annelids [16] and some arthropods [17] display temporal collinearity. That is, the order of the Hox genes along the chromosome also corresponds with the time of their first expression in the embryo, such that anterior genes are expressed earlier than any more posterior genes.

This review focuses on the role of Hox clusters in patterning the head–tail axis of bilaterians that develop in a regulative manner. Alternative, cell-lineage dependent (deterministic) development, such as seen, for example, in nematodes [18], and ascidians [19] is not addressed.

## 2. Seeking Sense in the Hox Gene Cluster

### 2.1. Seeking Sense in the Evolutionary Origin of Hox Clustering and Transcriptional Direction

Clustering arose due to gene duplication from an original proto-Hox gene. Duplication occurs due to unequal cross-over of DNA during meiosis in germ cells. This may be due to a cross-over between repeat elements in the DNA, or between other regions of a similar genetic sequence. It results in one chromosome becoming deleted in a genetic fragment—such as a Hox gene in our case—while its attached homologue incorporates the extra fragment.

This process, called “tandem gene duplication” leaves one chromosome with both the maternal- and paternal-derived copies of the Hox gene lying in tandem on the same DNA strand, and in the same orientation [20,21]. If this expanded chromosome contributes to a zygote, then the additional Hox copy offers new scope for evolutionary change. For example, if it acquires a new anterior boundary of expression and function, due to a change in regulation or mutation, then this can specify a new zone of development along the head–tail axis. It is usually suggested that this re-purposing of the new Hox gene occurs after the duplication event [21]. However, it has been proposed that two identical Hox genes may provide a selective disadvantage and a more reasonable hypothesis might be that the genes were initially alleles of the same gene that already possessed some useful differences in expression and/or function [22].

Repeated cycles of tandem gene duplication then led to the growing Hox gene cluster, permitting ever-increasing complexity of body structures along the head–tail axis. The process neatly explains not only why the Hox genes were formed in clusters, but also why they transcribe in the same direction. The cluster developed with spatial collinearity, and its polarity (that is, whether anterior-expressed genes lie upstream or downstream of posterior genes) was likely established with the initial duplication/re-purposing event [23].

Hox genes are members of the ANTP class of homeobox-containing genes. This also includes developmental gene groups paraHox, Dlx and NK, and all are thought to have arisen by tandem duplication from the same ANTP class proto-Hox gene [24]. Although paraHox, Dlx and NK genes are now usually dispersed from the Hox cluster some of these remain linked to the Hox cluster in at least some protostome and deuterostome species [24,25,26,27]. Apart from the ANTP class genes, there are ten other classes of homeobox genes [24], such as PRD, POU and LIM, and all classes likely arose by tandem gene duplication from an original proto-homeobox gene.

**Figure 4:**
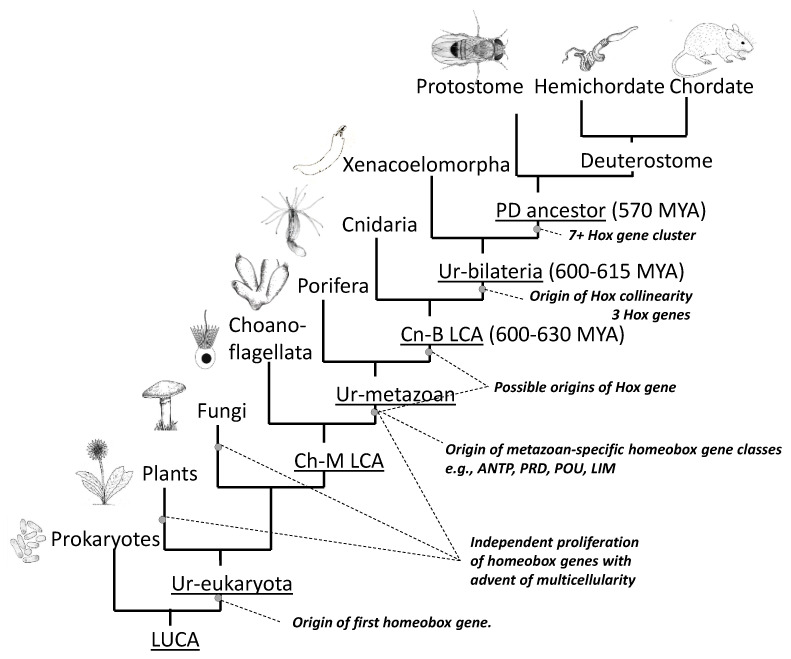
Origins of homeobox genes within the tree of life. Branching orders of Porifera relative to Cnidaria, and Xenacoelomorpha relative to protostomes and deuterostomes are uncertain. LUCA, last universal common ancestor; Ch-MLCA, choanoflagellate-metazoan last common ancestor; Cn-BLCA cnidarian–bilaterian last common ancestor; P-DLCA, protostome–deuterostome last common ancestor; MYA, million years ago. Figure from Gaunt 2019 [6].

The question then arises as to when these events took place. Figure 4 indicates the origin of homeobox genes within the tree of life. There are no ANTP class genes outside the metazoa [24]. However, duplication and diversification of ANTP class genes must have occurred very early in pre-bilaterian evolution because at least some poriferans (sponges) possess both NK [24] and paraHox genes [28] even though they lack Hox genes, which are presumed to have been secondarily lost [28]. Cnidarians possess Hox, paraHox and NK genes, but there is debate over whether cnidarian Hox genes are strict orthologues of bilaterian Hox genes, and whether they show spatial collinearity (Section 2.9). Proceeding backwards in time, the greatest proliferation of homeobox genes occurred with the advent of multicellularity in animals (metazoa), and also in plants and fungi (Figure 4) [29]. Bacteria do not have homeobox genes. However, they do have helix-loop-helix genes which have similarities in structure and function, and which may have been the ancestral progenitors of homeobox genes [29].

Overall, the distribution of homeobox and Hox genes throughout the tree of life makes sense in terms of the progressive development over time of the Hox gene cluster.

### 2.2. Seeking Sense in the Maintenance and Compactness of Clusters

Clusters are not always maintained. Some species may develop splits in the cluster, e.g., *Drosophila* where splits occur at different positions in separate sub-species [30]. However, the large regulatory regions between insect Hox genes probably limit the number of places where clusters can be broken without affecting gene function [31]. Other species, such as the urochordate *Oikiopleura*, show complete dispersal of the Hox cluster throughout the genome though, remarkably, the genes continue to show spatial collinearity in expression relative to the position that they occupied in the ancestral cluster [19]. 

Most species, however, continue to maintain the clustering of their Hox genes, at least to some extent. One explanation for this is enhancer sharing between Hox genes. For example, the *iab-5* regulatory region in *Drosophila* apparently regulates both *abd-A* and *Abd-B* [32]. Similarly, in mice, the CR3 enhancer regulates both *Hoxb4* and *Hoxb3* to become expressed up to the level of rhombomere 6/7 [33], and a separate Kreisler element then also drives *Hoxb3* expression in rhombomere 5 [34]. These are examples of local enhancer sharing, where the enhancer lies within the Hox gene cluster. 

**Figure 5:**
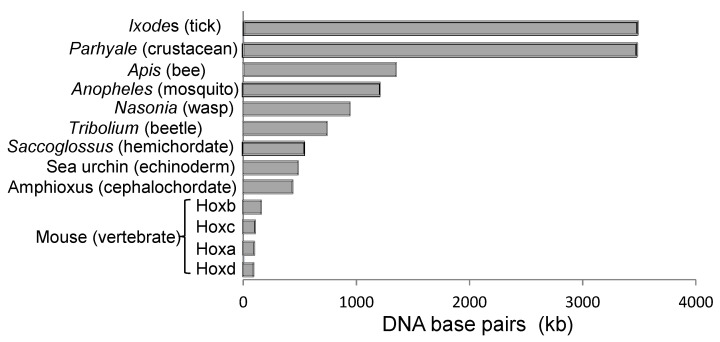
Hox clusters are more compact in vertebrates than in non-vertebrates. Clusters shown are all apparently intact. Sizes shown are from: *Ixodes* [37]; *Parhyale* [38]; *Apis* [39]; *Anopheles* [37]; *Nasonia* [37]; *Tribolium* [40]; *Saccoglossus* [41]; sea urchin [42]; amphioxus [36]; mouse [37].

In vertebrates, there are also long-range enhancers, usually located outside the Hox gene cluster, which can loop-in to regulate multiple Hox genes in a tissue-specific way [35,36]. Long-range enhancers, seen in vertebrates, encourage compaction and exclusion of other, non-Hox, genes. Compact clusters of vertebrates (Figure 5) are likely a derived rather than ancestral condition [36].

Other proposed reasons to maintain clustering are provided by the chromatin opening model, the Hox conjecture, both discussed below, and by the supposition that transcriptional activation and repression may each work best on nearby genes, as discussed in Section 2.3. Species that have lost their clustering have presumably overcome the above requirements.

Transcriptional direction within Hox clusters is usually maintained, but the *Dfd* gene in *Drosophila* provides an example of gene inversion from the ancestral state (Figure 1). Inversion is normally selected against because the promoter of the inverted gene may fall under the regulation of neighbour gene enhancers, with serious consequences for development. For example, the well-known dominant *Drosophila Antennapedia* homeotic mutation in which legs develop instead of antennae is usually due to inversions in the *Antp* gene, causing it to become ectopically expressed by the enhancers of upstream, more anteriorly-expressed genes [43,44]. Darbellay et al. [45] inverted mouse *Hoxd11* and *d12*, including displacement of a CTCF insulator element that normally separates them from *Hoxd13*. This caused an anterior shift in expression from the *Hoxd13* locus, possibly due to its misregulation from a *Hoxd11/d12*, or long-range, enhancer. These authors also describe a *Hoxd11* inversion that robustly suppressed expression from its *Hoxd12* neighbour, and they propose that this may be due to the collision of transcription units on opposing DNA strands.

### 2.3. Seeking Sense in Spatial and Temporal Collinearities

Spatial collinearity describes the correspondence between the order of genes along the cluster (3′ to 5′) and the order of their expression domains (anterior-to-posterior) along the developing embryo. Temporal collinearity describes a correspondence between the order of genes along the cluster (3′ to 5′) and the time of their first expression (early to late) in the embryo.

Many embryo types, including vertebrates, grow by successive addition of new parts at their posterior ends. That is, they develop in a head-to-tail temporal sequence from a posterior growth zone, and each new zone moves its overall Hox expression one step down in the pattern shown in Figure 2A, right. The “chromatin opening” model was developed principally for embryos that develop from a posterior growth zone. It proposes that genes are expressed by progressive, timed, 3′ to 5′ opening of Hox cluster chromatin structure that permits and regulates the expression of Hox genes in their anterior-to-posterior temporal sequence (temporal collinearity) [46]. This means that temporal collinearity specifies the need for, and is dependent upon, spatial collinearity. If this was so ancestrally, then we can likely predict that the ancestral bilaterian already had temporal collinearity [47] and developed its body axis from a posterior growth zone [48].

While it is clear that there is indeed a progressive opening of the Hox cluster as gastrulation proceeds [9], it is not obvious whether this is a cause or a consequence of Hox gene activation. Some experimental evidence against the chromatin opening model has been reviewed earlier [8]. More recent work in mouse embryonic stem cell aggregates [49] indicates that CTCF-binding insulator elements are successively breached along the Hoxd cluster, with accompanying, progressive chromatin extrusion and change in Hox gene expression. This process in itself suggests a need for some level of spatial collinearity and would be in keeping with the chromatin opening model. However, the authors note that spatial and temporal collinearities persist even after deletion of these insulator elements, and they conclude that insulators regulate the pace and precision of collinearities rather than their organization [49,50]. Duboule’s Hox Conjecture [51] proposes that for reasons yet unclear, but not necessarily dependent upon chromatin opening, spatial collinearity may still be necessary to achieve temporal collinearity in Hox expression. Evidence for this is largely circumstantial and is that species displaying temporal collinearity have so far been found to have an intact, unbroken Hox gene cluster. Studies on the annelid *Urechis unicinctus* perhaps provide some evidence against this [52]. Its posterior Hox genes (*Lox2* and *Post2*) are separated from more anterior genes (*Hox1* to *Lox4*) by greater than 1Mb, as sub-clusters, yet the genes as a whole display spatial and temporal collinearities [52]. Conversely, however, an intact cluster need not necessarily imply temporal collinearity. For example, the hemichordate *Saccoglossus* [41] has an intact cluster, but apparently does not show temporal collinearity [53].

An alternative “gene segregation” model [8] was proposed to account for why spatial collinearity is largely maintained even in species not showing either temporal collinearity or posterior growth zone, including perhaps the ancestral bilaterian. It could also be called the “single boundary” model. In this model, spatial collinearity is the primary feature and temporal collinearity follows as a necessary consequence in animals that develop from a posterior growth zone. This is so in these animals because anterior parts form first due to the earliest expression of anterior Hox genes, which are 3′-located. Posterior parts form last due to the late expression of posterior Hox genes, which are 5′-located. Overall, spatial collinearity thereby gives rise to temporal collinearity. The model does not dispute the importance of timing in Hox gene activation, especially so in species that develop from a posterior growth zone, but it does suggest that this may not depend primarily upon spatial collinearity, as it does in the chromatin opening model.

Comparison of Figure 2A with Figure 2B shows how spatial collinearity (Figure 2A) provides the minimum number (one) of boundaries between expressible and non-expressible Hox genes within the cluster at all positions along the body (Figure 3). The single boundary model proposes that the bilaterian cluster was established and maintained with spatial collinearity because this arrangement allows a number of potential benefits: see [8] and references therein, and now briefly summarized as follows. (i) Minimal boundaries mean the minimum risk that gene repressive factors, e.g., Pc protein, will inadvertently spread from inactive to active genes; and also, a minimum risk that gene activating factors, e.g., Trx protein, will spread from active to inactive genes. (ii) All active genes can more readily be grouped together in a nuclear transcription factory where there may be higher local concentrations of transcription and other factors necessary for gene expression. Additionally, all non-expressible genes can be grouped together in a nuclear Pc body where there may be higher local concentrations of repressive factors. (iii) Segregated active and inactive genes are less likely to interfere sterically with each other in their movement to transcription factories and Pc bodies. (iv) Contiguity of repressed Hox genes allows potential cross-spreading between them of repressive components.

Hox gene collinearity, well known to be ubiquitous in bilaterians, therefore remains an enigmatic feature of Hox gene expression. The ancestral bilaterian cluster was likely to have had an incompact Hox cluster, with vertebrate clusters having since become compacted [36]. Vertebrate Hox genes, perhaps becoming mutually more entangled in their regulation, might therefore not provide the ideal system to make sense of the ancestral state. There remains a need to investigate collinearity in a wide range of species.

### 2.4. Seeking Sense in the Partially Overlapping Nature of Hox Gene Expression Patterns

**Figure 6:**
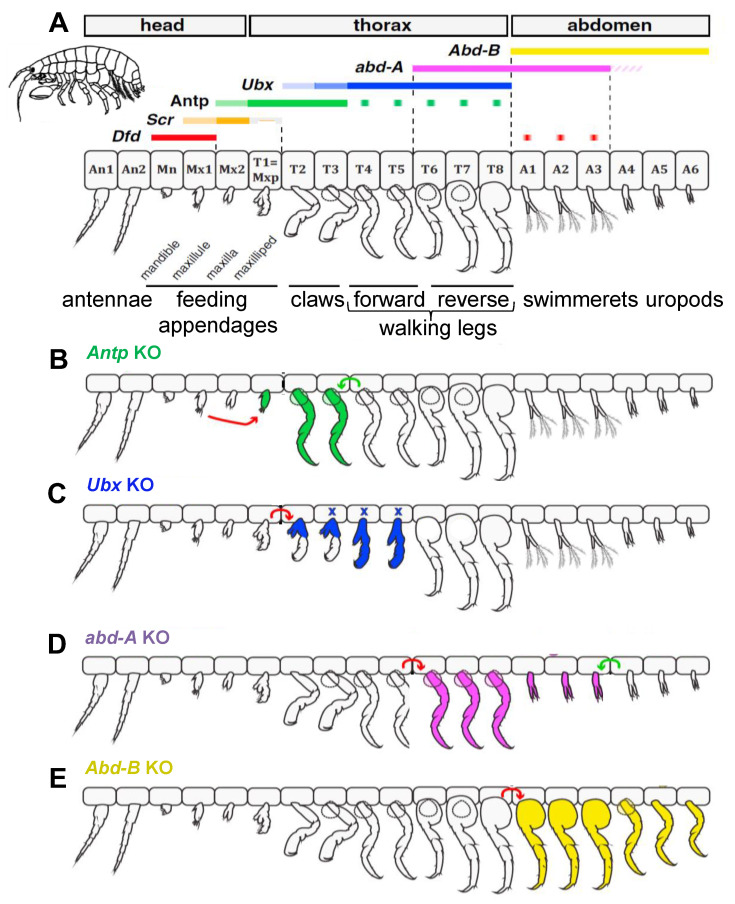
*Parhyale* Hox gene expression patterns in appendages and their knockout phenotypes. (**A**) normal Hox expressions and appendage phenotypes [17], (**B**–**E**) Hox gene knockout (KO) appendages: *Antp* (**B**); *Ubx* (**C**); *abd-A* (**D**); *Abd-B* (**E**). Arrows indicate directionality of the homeotic changes. Reproduced/modified from Martin et al. [54], with permission of Elsevier.

**Figure 7:**
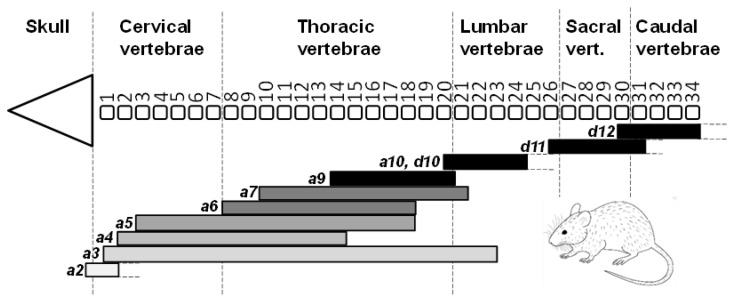
Mouse Hoxa and/or Hoxd expression domains within the pre-vertebral column of mid-gestation embryos. Hox shading intensities indicate orthologue groups, as in Figure 1. Expression patterns are as described in the following references: *Hoxa4-a10* [55]; *Hoxa2* [56]; *Hoxa3* [57]; *Hoxa10* [58]; *Hoxd10-d12* [14].

There are three obvious features of Hox gene activity (Figure 2A, Figure 6A and Figure 7). First, each gene is usually expressed over a long A-P domain, and for segmented animals this means that it is usually expressed across multiple segments. Second, within these long domains there is usually a gradation of Hox expression intensity, often forming an A-P gradient. Third, the long expression domains for different Hox genes are usually partially overlapping along the head–tail axis.

These features of Hox gene expression can greatly increase the number of possible positional addresses along the body. For example, a cluster of 13 Hox genes expressed without graded activity or overlap could provide only 14 positional addresses (if we assume that the most anterior address is the absence of Hox gene expression). However, if cells are able to monitor their intracellular concentrations of two or more Hox expressions then many more than 14 positional addresses become possible.

The long domains of Hox gene expression allow structural features to be reiterated along the body. For example, forward walking legs in the crustacean *Parhyale* occur on adjacent segments that express *Ubx* but not *abd-A* (Figure 6A). Regions along the body of arthropods that show similar anatomical features are called tagmata. A change from one tagma to the next is typically accompanied, and caused (Section 2.7 and Section 2.8), by a change in the complement of Hox gene activity.

Similarly, along the mouse vertebral column, a sudden change from one vertebral type to another is usually marked, and caused (Section 2.7), by the anterior expression boundary of a new Hox gene. For example, Hox6, Hox10 and Hox11 expression boundaries mark, respectively, the transitions from cervical to thoracic, thoracic to lumbar and lumbar to sacral vertebrae (Figure 7).

With an overlapping pattern of Hox gene expressions, the question then arises as to whether two or more Hox genes expressed in a region are cooperative (combinatorial) in their actions, or whether one is dominant (hierarchical) in its effect over the other(s). Some examples are known of combinatorial Hox gene activity. In *Parhyale*, for example, *abd-A* and *Abd-B* are expressed in partially overlapping domains along the abdominal segments (Figure 6A). Segments expressing *abd-A* in absence of *Abd-B* develop reverse walking legs; those expressing both *abd-A* and *Abd-B* develop swimmerets (also called pleopods); and those expressing only *Abd-B* develop uropods. Swimmerets apparently require combinatorial gene activity since animals mutant for either *abd-A* or *Abd-B* do not develop swimmerets (Figure 6D,E, Section 2.7). Although cooperating Hox genes are seen to be expressed in the same tissue it is not always clear whether or not they are also expressed in the same cells.

More common are examples of how a Hox gene is dominant in its developmental effect over any more anteriorly expressed Hox genes, a mechanism known as “posterior prevalence” [59,60]. Dominance may act at the level of suppressing a more anterior Hox gene’s expression, or only its function. The mechanisms are often unclear but at least some cases involve microRNAs or long non-coding RNAs [61]. In the *Pc* mutant of *Drosophila*, *Abd-B* expressed anterior to its normal domain is able to prevail in its function over those of all more anterior Hox genes, converting all segments to the morphology of the eighth abdominal segment (A8) [62]. *Drosophila Ubx* is normally expressed in parasegments 5 to 13 and notably acts to specify parasegment 6, visualized in A1. Over- and ectopic-expression of *Ubx* throughout the *Drosophila* embryo [63] results in the transformation of anterior segments towards an A1 morphology since *Ubx* function dominates over those of more anterior Hox genes. However, overexpression of *Ubx* in segments posterior to A1 does not affect their morphology since *abd-A* and *Abd-B* are dominant to *Ubx*. Many similar examples are seen in mice. For example, Hox10 genes suppress the rib-forming activity of more anteriorly expressed Hox genes in order to specify the rib-free lumbar region [64]. 

With an overlapping pattern of Hox gene expressions (Figure 2A) dominance of posterior genes makes sense: it allows for a sharp A-P boundary between one set of anatomical structures and the next. Crucially, it requires that posterior, dominant gene expression is excluded from anterior parts of the embryo, and this is the mechanism sustained by Pc repression (Figure 3).

### 2.5. Seeking Sense in the Head–Tail Polarity of Hox Expression Domains

**Figure 8:**
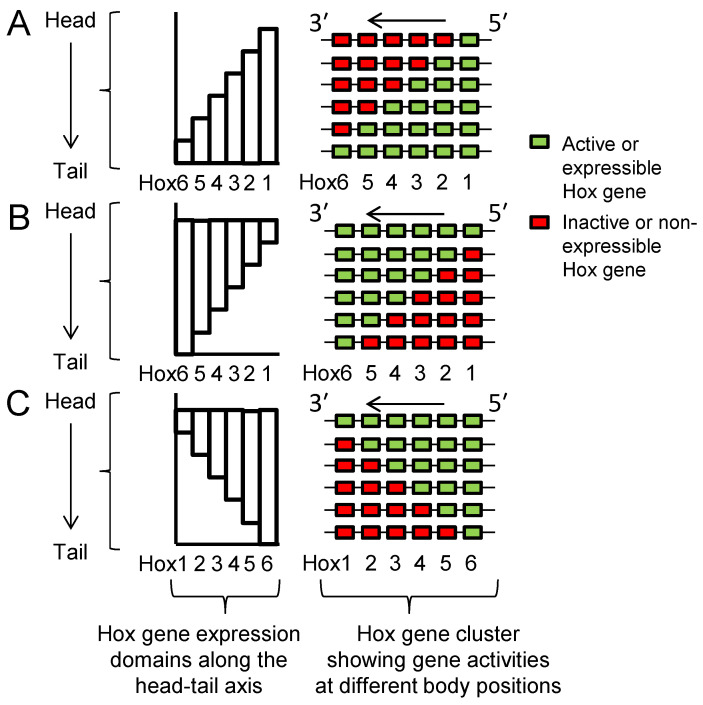
Potential head-to-tail Hox gene expression polarities as alternatives to that found in nature (Figure 2A). Each alternative (**A**–**C**) displays its own form of spatial collinearity. Arrows show directions of transcription.

A partially overlapping pattern of Hox gene expression could be formed with spatial collinearity in the pattern shown in Figure 2A, but also as the alternative forms of Figure 8A–C. In scenario 8A the Hox genes lie in the opposite 3′ to 5′ relative positions from that found in nature (Figure 2A). While this may appear to be acceptable for embryo development it is also possible that it is less so due to read-through into more posterior, and dominant, Hox genes. Transcriptional read-through in the Figure 2A cluster can result only in sense transcripts for more anterior, and likely therefore repressed, Hox genes. There are several reports of transcriptional read-through between adjacent Hox genes: in crustaceans [65], in human *HOXC6*, *C5* and *C4,* where the same 5′ non-coding exon is spliced on both *HOXC6* and *HOXC4* mature placental mRNAs [66], and in several other mammalian Hox genes where the function is unknown [67]. 

Scenarios 8B and 8C could work only if anterior (Hox1-end) genes had evolved dominant to posterior genes. In animals that develop in a head-to-tail temporal sequence there would be a need for sequential suppression of Hox genes and their proteins. Sequential removal of Hox proteins from cells may be less easily controlled than their sequential activation, as is the case in Figure 2A. The fact that the cluster did not arise in the form of Figure 8B or Figure 8C may be support for the suggestion that the ancestral animal did indeed develop in a head-to-tail temporal sequence [48]. Scenario 8C carries the possible additional problem of read-through into more dominant (this time, anterior) Hox genes.

These arguments suggest that there is sense in the fact that the Hox cluster developed with the head-tail polarity of Figure 2A. However, we know that the Hox cluster evolved successfully only once, and it may have been by chance alone that this was in the way shown in Figure 2A.

### 2.6. Seeking Sense in the Duplication of Hox Genes and Clusters

An increase in the number of genes within the cluster potentially provides more positional cues along the body. For example, one ancestral Hox gene duplicated to give *Antp*, *Ubx* and *abd-A* in *Drosophila* and, independently, Hox gene groups 6 to 8 in vertebrates (Figure 1). These genes have now acquired different roles and expressions (Section 2.7 and Section 2.8). Similarly, the ancestral *Abd-B* gene was duplicated to give Hox gene groups 9 to 13 in vertebrates and some other deuterostomes (Figure 1). Most protostomes, however, acquired diversity without substantial change in Hox gene numbers [5,6].

A more profound increase in Hox genes has been achieved in vertebrates by whole cluster duplications. Two rounds (2R) of whole genome duplication, likely occurring by chance in early vertebrates, gave rise to the four clusters seen today in mice and humans [68]. Analysis of the Hox genes present or missing in the four clusters indicates that the A and B and the C and D clusters are most likely to be sister clusters. That is, the 1R event resulted in two clusters (A,B) and (C,D). The 2R event then resulted in four clusters A, B, C and D [68]. Modern bony fish often have more than 4 clusters due to ancestral 3R and 4R events [69]. 

Several reasons can be suggested as to why cluster duplication was advantageous in vertebrate evolution. First, it permitted increased complexity along the head-tail axis. Thus, while expression studies [70] and functional analyses (Section 2.7) have revealed some cases where paralogous genes from different mouse clusters apparently share the same or similar anterior boundaries of expression and function (e.g., *Hoxa4, b4, d4*), in other cases paralogues have clearly adopted different expression boundaries (e.g., *Hoxc4*), suggesting that duplication could have provided opportunity for increased positional information along the head-tail axis [57]. 

Second, cluster duplication allowed individual clusters to acquire separate roles. This is suggested by observations that expression in developing spinal cord tracts and in internal organs is often similar for genes within a cluster but different for genes between clusters. As examples, Hoxb genes alone are confined in expression to the dorsal nerve tracts of the mouse embryo spinal cord [57]; Hoxc genes (*c4* and *c5*) are expressed abundantly in the embryonic mouse oesophagus but not the trachea, while Hoxa genes (*a3*, *a4*, *a5*) are expressed in trachea but not oesophagus [71,72]. Similarly, Hoxc (*c5*, *c6*) and Hoxd (*d4*, *d8*, *d9*) genes are expressed in the embryonic mouse testis [73,74], while Hoxa and Hoxb genes are not. 

Third, cluster duplication may have been required in other ways for anatomical change in part of the vertebrate body. For example, heart tube folding and Hox cluster duplication are features present in vertebrates but not in non-vertebrate chordates. Heart folding required duplication to Hoxa and Hoxb clusters, and deletion of either from mice results in a heart tube that does not fold: an apparent atavistic change [75]. It is not certain whether heart folding is due to a gene dosage effect or to an acquired, cooperative, regulatory difference between the Hoxa and Hoxb clusters.

The question arises as to why cluster duplications have provided a selective advantage in vertebrates but not, typically, in non-vertebrate deuterostomes or most protostomes. Vertebrates are believed to be more constrained than other animals in the numbers and spacing of the Hox genes and regulatory elements within their clusters (Figure 5). This is likely due to their acquired dependence on long-range enhancer elements which co-regulate multiple contiguous genes within each cluster (Section 2.2). Such regulations are correlated with vertebrate-specific traits [36,76,77]. In vertebrates, therefore, it is likely that the numbers of Hox genes could increase further only by whole cluster duplications [78].

Just as evolving increased axial complexity has demanded rising numbers of Hox genes, so evolving increased axial simplicity may be accompanied by loss of Hox genes. For example, tardigrades are protostomes, and ecdysozoans, that possess only four pairs of legs due to the apparent loss from the ancestral condition of more posterior leg-bearing segments. The Hox genes that specified the lost segments (*Antp, Ubx* and *abd-A*) are now absent from the tardigrade genome [79]. A similar example might be the acoel flatworms which are bilaterians possessing only three Hox genes that represent the anterior, middle and posterior groups of other bilaterians [80,81]. Acoels are sometimes regarded as degenerate deuterostomes [82,83]. A more intriguing view, however, is that acoels are primitive bilaterians that form part of a sister group to all other bilaterians [84]. Consistent with this, all members of this group lack features that are common to most other bilaterians such as an anus, kidney tubules and a circulatory system. The acoels might therefore be providing us with a glimpse into the early evolution of the bilaterian Hox gene cluster.

### 2.7. Seeking Sense in the Phenotype of Hox Gene Knockouts

Reference to Figure 2A shows how partially overlapping domains of Hox expression along the body dictate that loss (e.g., by knockout) of a Hox gene results in its functional domain expressing the Hox profile normally expressed by the more anterior segment(s). In consequence, this region commonly duplicates the anatomy of a more anterior segment (producing a so-called anterior homeotic transformation). This is a prediction intrinsic to Lewis’s model. For example, the *bxd* mutation in *Drosophila* disrupts a normal pattern of *Ubx* expression in parasegment 6 [85,86]. In keeping with this, the *bxd* mutant has a pair of legs on the first abdominal segment, now specified by the *Antp* gene. As described below these same principles are seen in the results of Hox gene knockout studies in another arthropod, *Parhyale*, and in vertebrates.

In *Parhyale* (Figure 6A), a crustacean, the T1 segment bears a pair of maxillipeds (feeding appendages, such as the maxillae on head segments); T2–T3 bear prehensile claw-like chelipeds (legs bearing claws); T4–T5 bear forward walking legs; T6–T8 bear reverse walking legs; A1–A3 bear swimmerets; and A4–A6 bear uropods. Each of these morphologies corresponds with its own unique Hox expression profile as follows. (1) The maxillipeds of T1 express *Antp* but not *Ubx*. (2) The chelipeds of T2 and T3 express high levels of *Antp* but only low levels of *Ubx*. (3) The forward walking legs of T4 to T5 express high levels of *Ubx*, while the reverse walking legs on T6-T8 additionally express *abd-A*. (4) The swimmerets on A1–A3 express both *abd-A* and *Abd-B*. (5) The uropods on A4–A6 express only *Abd-B*.

Knockout of Hox genes in *Parhyale* [54] generally supports the predictions of Lewis’s model (Figure 2A). Knockout of *Ubx* results in partial transition of cheliped appendages on T2 and T3 to maxilliped morphology (Figure 6C), presumably under the influence of *Antp*. Knockout of *abd-A* results in only one leg type, forward walking leg, presumably formed under the influence of *Ubx* (Figure 6D). Knockout of *Abd-B* transforms swimmerets into reverse walking legs, presumably under influence of *abd-A*; and uropods into forward walking legs, perhaps due to loss of a normal *Abd-B* posterior prevalence over *Ubx* in the posterior segments (Figure 6E). In apparent contradiction to Lewis’s model, knockout of *Antp* in *Parhyale* transforms T2 and T3 chelipeds into forward walking legs, a posterior homeotic transformation (Figure 6B). This could be explained if the low levels of *Ubx* expression in T2 and T3 are normally overridden by high levels of *Antp* (Figure 6A).

**Figure 9:**
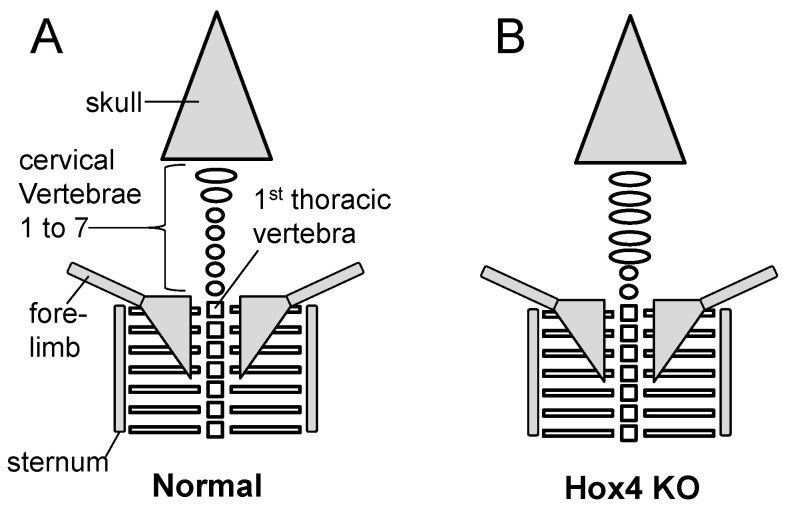
Mouse knockout (KO) anterior skeletal phenotype for Hox4 genes. (**A**) Normal newborn mouse. (**B**) Mouse knocked out for six Hox4 genes (Hoxa4, b4, d4, maternal and paternal).

Knockout of Hox genes in mouse generally supports the predictions of Lewis’s model (Figure 2A). *Hoxa4, b4* and *d4* paralogues all share an anterior boundary of expression at the second cervical vertebra, C2 or axis (Figure 7) [70]. In the absence of *Hoxa4, b4* and *d4* (that is, absence of all six genes, both maternal and paternal copies) the first five cervical vertebrae assume a complete or partial C1 identity (Figure 9), presumably under the influence of Hox3 genes. The extent of this transformation varies with the dose of normal Hox4 genes [87]. Thus, in single mutants for *Hoxb4* or *Hoxd4* there is sometimes a partial transformation of C2 to C1 morphology. In *Hoxb4/Hoxd4* double mutants C2 is almost completely transformed to a C1 identity, but if double mutants possess only a single copy of a normal *Hoxb4* or *Hoxd4* gene then the C2 to C1 transformation is less complete.

**Figure 10:**
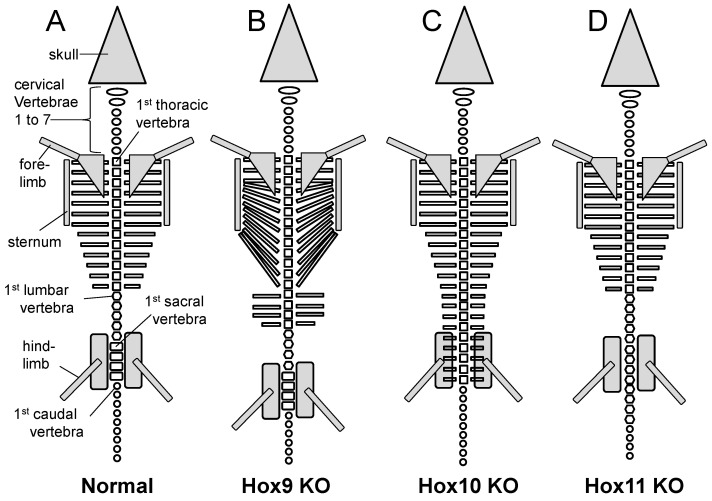
Mouse knockout (KO) skeletal phenotypes for Hox9, Hox10 and Hox11 genes. (**A**) Normal newborn mouse. (**B**) Mouse knocked out for all eight Hox9 genes (a9, b9, c9, d9, maternal and paternal). (**C**) Mouse knocked out for all six Hox10 genes (a10, c10, d10, maternal and paternal). (**D**) Mouse knocked out for all six Hox11 genes (a11, c11, d11, maternal and paternal). Figure from Gaunt [6].

The Hox9 group of mouse Hox genes, expressed as in Figure 7, has its main zone of influence in the posterior thoracic region. In the normal embryo, the first seven pairs of ribs attach to the sternum ventrally and the next six pairs attach either to the seventh pair or are unattached ventrally (Figure 10A). It appears that Hox9 genes are required for the formation of non-sternal ribs. Thus, in absence of all eight Hox9 genes (that is, both maternal and paternal copies) the posterior thoracic vertebrae fall under the influence of the more anterior Hox genes resulting in about 13 pairs of ribs attached to the sternum (Figure 10B) [88]. In addition, thoracic-type vertebrae with unattached ribs replace the anterior lumbar vertebrae. As found for Hox4 genes the most severe phenotypes require extensive loss of normal genes within the Hox9 group. Leaving just one normal Hox9 gene in a mutant that is otherwise mutant for seven Hox9 genes results in a less severe phenotype.

The Hox10 group of mouse Hox genes, expressed as in Figure 7, has its main influence in the lumbar region. This apparently requires suppression of more anterior Hox genes that fashion the thoracic (ribbed) phenotype. In absence of all six Hox10 genes (Hoxa10, c10, d10) [89], the lumbar and sacral regions fall under the influence of more anterior Hox genes resulting in thoracic type vertebrae with ribs instead of normal lumbar and sacral vertebrae (Figure 10C). The presence of only one functional Hox10 gene results in a much less severe phenotype, displaying only one extra pair of ribs.

The Hox11 group of genes, expressed as in Figure 7, has its main influence in the sacral region. This apparently requires suppression of more anterior genes that fashion the lumbar phenotype. In absence of all six Hox11 genes (Hoxa11, c11, d11) [89], the lumbar and sacral regions fall under the influence of more anterior Hox genes resulting in lumbar type vertebrae instead of normal sacral vertebrae. The lumbar-type vertebrae also replace the most anterior caudal vertebrae (Figure 10D). The presence of only one functional Hox11 gene results in a less severe phenotype.

### 2.8. Seeking Sense in the Evolution of Axial Morphologies by Shifts in Hox Expression

**Figure 11:**
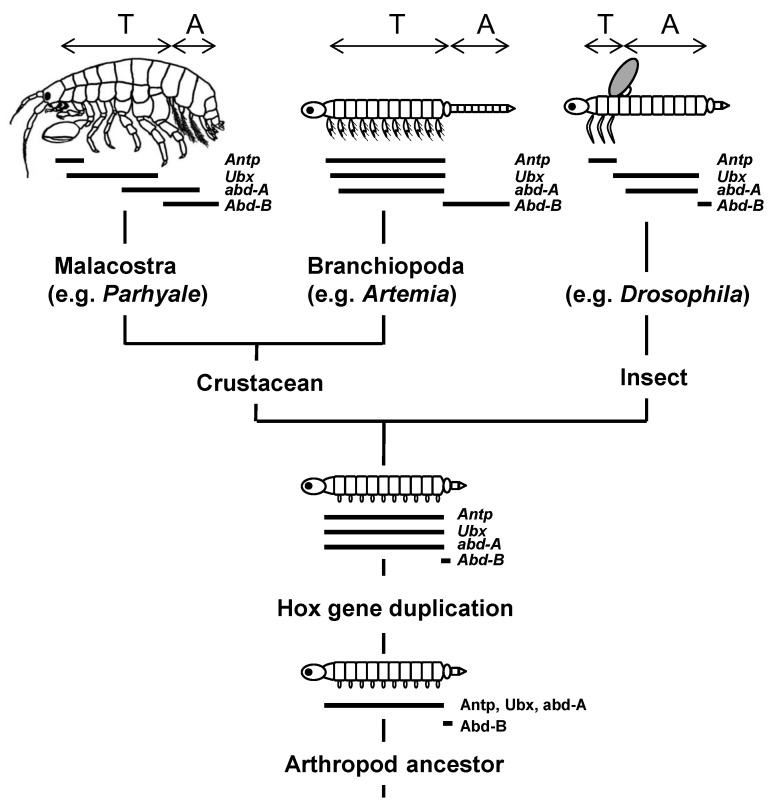
Hox expressions in arthropods. Figure drawn mainly from Abzhanov and Kaufman [94], Averof and Akam [91], Serano et al. [17] and McCarthay-Taylor et al. [92]. T, thorax; A, abdomen. Figure from Gaunt 2019 [6].

We have already mentioned two ways that may lead on to an evolved change in axial patterning. First, the generation of new Hox genes by duplication of either individual genes or entire clusters. These new genes may then acquire new expressions and functions (Section 2.1 and Section 2.7). Second, acquisition in vertebrates of long-range enhancers located outside the ancestral Hox cluster (Section 2.2). These allow the use of the Hox cluster, in addition to its role in head–tail patterning, to specify development along evolutionarily novel structures such as limbs and external genitalia [35]. However, the main objective in what follows is to examine the commonly utilized mechanism whereby axial morphologies may change by shifts in the expression domains of existing Hox genes, without the prior need to generate new ones. The mechanism is especially clear in arthropods where axial patterning is highly variable, and is seen readily in the range of appendage types, even though the overall complement of Hox genes remains rather stable [37]. Figure 11 shows the expression domains of a variety of Hox genes in arthropods *Drosophila*, *Artemia* and *Parhyale*.

In insects, *Abd-B* is expressed in the posterior-most segments of the abdomen [90] and *abd-A* is not expressed in the thorax [91]. In crustaceans, *Abd-B* is expressed throughout the abdomen and *abd-A* expression extends into the thorax [17,91,92]. It has been suggested that the pattern of *Ubx, abd-A* and *Abd-B* expressions in ancestral arthropods may have been similar to those found today in *Artemia* [91] (Figure 11).

There is also variation between arthropod types in which of the Hox genes *Ubx, abd-A* and *Abd-B* are able to suppress limb development [92]. This may have occurred in a step-wise sequence over time, and it takes place most notably by their suppression of the *Distalless (Dll)* gene [93]. In *Parhyale* (Malacostra) none of these genes repress limb development. In *Artemia* (Brachiopoda) it has been suggested that only *Abd-B* represses limb development [92]. In *Drosophila* (Diptera) *Ubx, abd-A* and *Abd-B* all suppress limb development.

How may the ancestral arthropod have evolved the diverse head–tail morphologies seen among arthropods today, bearing in mind the evolved changes in the roles of *Ubx, abd-A* and *Abd-B* as suppressors of *Dll* and limb development? Some likely possibilities are seen in Figure 6 and Figure 11. First, in the ancestry of *Drosophila* a backward shift of *Ubx* may have facilitated the formation of the three leg-bearing thoracic segments under the control of *Antp* (Figure 11). Second, a backward shift of *Ubx* in a *Parhyale* ancestor may have permitted activity of *Antp* in the anterior thorax, facilitating here the evolution of maxillipeds. On this topic, different crustacean species vary in the number of anterior thoracic segments where walking legs have become evolutionarily transformed into maxillipeds. An examination of 13 different crustacean species, where maxilliped numbers vary from 0 to 3 pairs, showed that changes in their anterior expression patterns of *Ubx* and *abd-A* correlate well with the axial level of the transition from walking leg to maxilliped [95]. Third, in the ancestry of *Parhyale* a backward shift in the anterior boundary of *abd-A* expression may have permitted the development of forward walking legs, under the control of the *Ubx* gene (Figure 6 and Figure 11). The Malacostra include the group Amphipoda (“different foot”) which has diverse walking leg types (e.g., *Parhyale*), but also the group Isopoda (“equal foot”) which typically has only one type of thoracic leg. In isopods, but not amphipods (Figure 11), *abd-A* extends over most of the thorax [94].

Vertebrate shifts in Hox expression, with corresponding morphological changes, have been shown most clearly in comparisons of mouse, chicken and goose. The position of the cervical/thoracic vertebral junction, and hence the length of the neck, has changed by axial shifts in expressions of Hox6 genes, known to regulate thoracic vertebra and rib development [96,97,98]. In an additional strategy, the rib cage of snakes extends posteriorly because Hox10 genes have lost their ability to block the thoracic phenotype. This is due to a mutation in the *Myf5* gene enhancer, which is normally a downstream target of Hox10 [99].

Most of the conclusions reached above regarding the effects of evolutionary shifts in Hox expression are supported by the results of Hox gene knockout studies (Section 2.7).

### 2.9. Seeking Sense in the Hox Clustering of Pre-Bilaterians

This article so far describes Hox gene clusters of bilaterally symmetrical animals (the Bilateria). Amongst pre-bilaterians, only Cnidaria possess Hox genes. These may include Hox genes orthologous with those of Bilateria: a group 1, a group 2, and a middle or posterior group Hox gene [100,101], suggesting that the bilaterian Hox cluster had already begun to form in the cnidarian/bilaterian ancestor, perhaps even to pattern its head-tail axis [102]. However, some authors question such close homology [103]. In the coral *Acropora*, all three Hox genes are linked in a single cluster, but in some other cnidarian species there is either no cluster or only clustering of the anterior genes [101]. The number of Hox genes often varies between different cnidarian species, and the particular genes present may also vary. Hox expression analyses on several cnidarians have shown no consistent evidence for collinearity, no consistency in expression patterns of orthologous Hox genes in different species, and therefore no evidence for a conserved cnidarian Hox code [100,103,104].

We can assume that our distant P-DLC bilaterian ancestor arose on an evolutionary pathway that had provided it with the core Hox gene cluster, collinear Hox expressions and, probably, a role for these expressions to specify pattern formation along its head–tail axis. It seems likely that alongside this ancestor other creatures were also experimenting with Hox genes, using them to specify body parts, and evolving ever more complex clusters. It is also likely that many of these were evolving Hox clusters with collinearity and so this alone seems unlikely to be the reason why one animal came to dominate and take over the world. Some other evolutionary event, perhaps the acquisition of a three-layered body wall, or bilateral symmetry, may instead have provided the selective advantage to our ancestor which allowed it to prevail. The cnidarians with their two-layered body wall and radial symmetry might therefore represent animals “stuck” at an early experimental stage of Hox cluster evolution.

## 3. Conclusions

It is suggested here that there is much sense in the way that the Hox cluster of bilaterians arose in clusters by gene duplication, expressed its genes in overlapping patterns with a specific head–tail polarity, maintained its clustered Hox genes with the same directions of transcription, shifted its expressions for change in head–tail patterning, generates anterior homeotic shifts in Hox gene knockout studies, and was compacted and duplicated during vertebrate evolution.

Hox gene collinearity, well known to be ubiquitous in bilaterians, remains an enigmatic feature of Hox gene expression. Two different models that attempt to make sense of it are discussed.

## Data Availability

Not applicable.
